# National Trends and Predictors of Adverse Outcomes in Hypoglycemia Hospitalizations in the United States, 2017–2022

**DOI:** 10.1002/edm2.70370

**Published:** 2026-10-22

**Authors:** Abu Huraira Bin Gulzar, Sheraz Hakeem, Muhammad Umer Sohail, Aima Tahir, Tamseer Tariq, Rasheeda Shehroz, Moiz Shah Khan, Ismail Rahim, Umme Aiman, Muhammad Afnan Ashraf, Shanzey Rai, Marriam Mavora, Asad Ali Ahmed Cheema, Hina Nadeem

**Affiliations:** ^1^ Department of Medicine Services Institute of Medical Sciences Lahore Pakistan; ^2^ Department of Medicine Aga Khan University Medical College Karachi Pakistan; ^3^ Department of Medicine Dow University of Health Sciences Karachi Pakistan; ^4^ Department of Medicine Akhtar Saeed Medical College Lahore Pakistan; ^5^ Department of Medicine Karachi Medical and Dental College Karachi Pakistan; ^6^ Department of Medicine Liaquat University of Medical and Health Sciences Jamshoro Pakistan; ^7^ Department of Medicine Ziauddin University Karachi Pakistan; ^8^ Department of Medicine Dow Medical College Karachi Pakistan; ^9^ Department of Medicine Bhaskar Medical College Hyderabad Telangana India; ^10^ Department of Medicine Sharif Medical and Dental College Lahore Pakistan; ^11^ Department of Medicine Islamic International Medical College Rawalpindi Pakistan; ^12^ Department of Medicine Allama Iqbal Medical College Lahore Pakistan; ^13^ Department of Medicine, International School of Medicine International University of Kyrgyzstan Bishkek Kyrgyzstan; ^14^ Department of Medicine Jinnah Sindh Medical University Karachi Pakistan

**Keywords:** healthcare costs, hospitalization, hypoglycemia, in‐hospital mortality, National inpatient sample

## Abstract

**Objective:**

To assess national trends and inpatient outcomes among adults hospitalized with a primary diagnosis of hypoglycemia in the United States from 2017 to 2022.

**Methods:**

We conducted a retrospective cross‐sectional analysis of the National Inpatient Sample. Adult hospitalizations aged 18 years or older with a principal ICD‐10‐CM diagnosis of hypoglycemia were included. The annual share of primary hypoglycemia hospitalizations was calculated per 100,000 adult NIS discharges. Outcomes included in‐hospital mortality, length of stay(LOS), and inflation‐adjusted hospital cost standardized to 2022 US dollars. Multivariable survey‐weighted logistic regression was used for mortality, a survey‐weighted generalized linear model (GLM) with a quasi‐Poisson distribution and log link was used for LOS, and a survey‐weighted GLM with a gamma distribution and log link was used for inflation‐adjusted hospital cost.

**Results:**

The analysis included 330,910 weighted hypoglycemia hospitalizations. Mean age was 67 years; 52% were women; and 70% were Medicare‐insured. Diabetes mellitus (91%) was the most common comorbidity, while obesity (17%) was least common. Primary‐hypoglycemia discharges peaked in 2019 (195 per 100,000 adult NIS discharges), declined in 2021 (180), and increased again in 2022 (190). Mortality occurred in 1.6%, and median LOS was 3 days. Compared with 2017, adjusted mortality odds were higher in 2020 (OR 1.30, 95% CI 1.04–1.61), 2021 (OR 1.44, 95% CI 1.16–1.79), and 2022 (OR 1.33, 95% CI 1.07–1.65). Similarly, LOS and hospital costs were also higher in 2020, 2021, and 2022 compared to 2017. Congestive heart failure (CHF) and AF were associated with higher mortality odds, LOS, and cost.

**Conclusion:**

Primary hypoglycemia hospitalizations declined during the pandemic years but became more severe and resource‐intensive over time. Patients with CHF or AF had higher mortality, higher LOS, and higher hospital costs, highlighting the need for integrated hypoglycemia surveillance and cardiometabolic care.

## Graphical abstract

Among 330,910 weighted US hospitalisations for primary hypoglycemia from 2017 to 2022, discharge shares declined during the pandemic, while mortality, length of stay, and costs increased. Patients with congestive heart failure or atrial fibrillation had worse inpatient outcomes, supporting integrated hypoglycemia surveillance and cardiometabolic care.

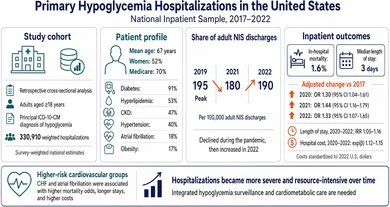

## 1 Introduction

Hypoglycemia is defined as a state when blood glucose levels drop below 70 mg/dL, marked by sweating, palpitations, tremors, hunger, confusion, or dizziness; however, most of the physical symptoms only appear when the glucose is below 55 mg/dL [1, 2]. Clinically, the diagnosis is usually confirmed via Whipple's triad, which includes the evaluation of blood glucose, followed by the identification of the signs and symptoms, and finally resolving the symptoms by correcting the blood sugar [1, 3]. From an epidemiologic standpoint, there is no direct evidence quantifying the annual hypoglycemia admissions; however, studies exist that explain the trends in parallel with associated diseases. For example, a study done on 512,261 patients with Type 2 diabetes (T2DM) showed that hospitalizations due to hypoglycemia decreased between 2016 and 2022; however, discharge diagnosis has increased over time [4]. Similarly, another study found that even though hypoglycemic emergencies improved for T2DM, the situation worsened for patients with Type 1 diabetes mellitus (T1DM) [5]. Despite these mixed trends, hypoglycemia remains a frequent cause of hospital admissions among US adults, posing a burden on the healthcare system by consuming valuable resources and increasing hospital costs [6]. Regarding risk factors, a study done on 201,705 adults back in 2020 deduced that major factors driving hypoglycemia included T1DM, multiple comorbidities, prior severe hypoglycemia, and use of glucose lowering drugs [7]. Over time, studies report an increasing incidence of hospitalizations in the US due to diabetes [8], chronic kidney disease (CKD) [9, 10], liver diseases [11] and heart failure (HF) [12]. Taken together, this poses a likely threat towards indirectly increasing the burden of hypoglycemia cases in the USA.

Despite this, hypoglycemia is a preventable complication. However, being associated with chronic conditions like alcoholic‐related liver diseases, liver failure, heart failure, endocrine dysfunction, diabetes, and CKD adds complexity to its control. Current programs, whether operating locally or nationally, serve to capture these diseases separately [13, 14, 15, 16, 17]. However, none of them directly answers contemporary national hospitalizations due to hypoglycemia across the strata of these diseases, which can help to determine the most contributing diseases and quantify hospitalization costs. Therefore, our study addresses this problem by analysing the most recent available national inpatient sample (NIS) from 2017 to 2022 and assesses the national trends, patient characteristics, comorbid disease patterns, and inpatient outcomes among US adults hospitalized with a primary diagnosis of hypoglycemia.

## 2 Methods

### 2.1 Study Design and Data Source

We conducted a retrospective, cross‐sectional analysis of adult hospitalizations using the NIS from 2017 through 2022. The NIS is developed as part of the Healthcare Cost and Utilization Project (HCUP), sponsored by the Agency for Healthcare Research and Quality (AHRQ). It is the largest publicly available all‐payer inpatient database in the United States and is designed to generate national and regional estimates of inpatient utilization, access, cost, quality, and outcomes. Beginning with the 2012 redesign, the NIS approximates a 20% stratified sample of discharges from US community hospitals, excluding rehabilitation and long‐term acute care hospitals. Unweighted, the NIS contains discharge‐level data from about 7 million hospital stays each year, and after applying discharge weights, it estimates more than 33 million hospitalizations nationally.

The study followed the Strengthening the Reporting of Observational Studies in Epidemiology (STROBE) recommendations for observational studies. Because the NIS is a de‐identified limited dataset, individual informed consent was not required.

### 2.2 Study Population

We included all adult inpatient admissions aged 18 years or older with a principal diagnosis of hypoglycemia between January 1, 2017, and December 31, 2022. We used the principal diagnosis field to identify admissions in which hypoglycemia was the primary reason for hospitalization. Hypoglycemia hospitalizations were identified using the following ICD‐10‐CM codes: E16.0, E16.1, E16.2, E10.641, E10.649, E11.641, E11.649, E13.641, and E13.649. These codes captured drug‐induced hypoglycemia, other or unspecified hypoglycemia, and diabetes‐related hypoglycemia with or without coma. The final weighted cohort included 330,910 adult hospitalizations with a primary diagnosis of hypoglycemia.

Because the NIS is discharge‐level rather than patient‐level data, the unit of analysis was the hospitalization. Therefore, repeated admissions by the same individual could not be linked across hospitalizations.

### 2.3 Exposure and Covariates

We extracted demographic, hospital‐level, and clinical covariates. Demographic variables included age, sex, race, median household income quartile by patient ZIP code, expected primary payer, and residence category. Race was classified as White, Black, Hispanic, Asian or Pacific Islander, Native American, and Other. Median household income was categorized into national quartiles, with the first quartile representing the lowest‐income ZIP code areas. Expected primary payer was categorized as Medicaid, Medicare, private insurance, and other payer.

Hospital‐level variables included hospital region, hospital bed size, and hospital location/teaching status. Hospital region was classified as Midwest, Northeast, South, and West. Hospital bed size was categorized as small, medium, or large according to HCUP hospital definitions. Hospital location and teaching status were categorized as rural, urban non‐teaching, and urban teaching. Residence type was categorized as large metropolitan, small metropolitan, and micropolitan. Clinical comorbidities were identified using ICD‐10‐CM diagnosis codes available in the hospitalization record. The selected comorbidities included CKD, congestive heart failure (CHF), iron deficiency anaemia, T2DM, smoking, hypertension, alcohol abuse, HIV, previous myocardial infarction, atrial fibrillation (AF), obesity, and hyperlipidemia. These conditions were selected because they may influence hypoglycemia risk, physiologic reserve, inpatient mortality, length of stay (LOS), and hospital cost [7].

### 2.4 Outcomes

The primary outcome was in‐hospital mortality, defined using the NIS discharge disposition mortality indicator. Secondary outcomes were LOS and inflation‐adjusted total hospital cost. LOS was measured in days using the NIS length‐of‐stay variable.

Hospital costs were estimated from total hospital charges by applying HCUP cost‐to‐charge ratio files. HCUP notes that charges represent the amount billed by the hospital, whereas costs estimate the actual resources used to provide care, including wages, supplies, and utilities. Cost‐to‐charge ratio files include variables that allow researchers to merge cost ratios with HCUP inpatient databases and convert hospital charges into estimated hospital costs. All cost estimates were inflation‐adjusted and standardized to 2022 US dollars.

We also calculated annual primary hypoglycemia discharges per 100,000 adult NIS discharges. For each year, the numerator was the weighted number of adult admissions with a principal diagnosis of hypoglycemia, and the denominator was the weighted number of all adult NIS discharges in that year. The rate was then multiplied by 100,000. This measure reflects the relative share of hospitalizations attributable to primary hypoglycemia rather than a population‐based incidence rate.

### 2.5 Statistical Analysis

All analyses accounted for the complex survey design of the NIS. We used discharge weights, strata, and hospital clustering to generate nationally representative estimates and correct standard errors. HCUP recommends applying discharge weights for national estimates. We summarized baseline characteristics using survey‐weighted descriptive statistics. Continuous variables were reported as mean with standard deviation or median with interquartile range, depending on distribution. Categorical variables were reported as weighted counts and percentages. Annual trends in primary hypoglycemia discharges, inpatient mortality, LOS, and inflation‐adjusted hospital costs were evaluated across calendar years.

We used survey‐weighted multivariable logistic regression to identify factors associated with in‐hospital mortality. Results from the mortality model were reported as adjusted odds ratios (OR) with 95% confidence intervals (CIs). We used negative binomial regression to evaluate factors associated with LOS; however, the stratified survey design of the NIS does not natively support a direct negative binomial family; therefore, we utilized a survey‐weighted generalized linear model (GLM) with a quasipoisson distribution and a log link. This model is mathematically rigorous and accounts for the overdispersed count data. Moreover, for the inflation‐adjusted hospital cost, we utilized survey‐weighted GLM with a gamma distribution and a log link. Results for hospital adjusted costs were reported as exp.(beta) coefficients with 95% CIs. An exp.(β) greater than 1 represented a higher adjusted hospital cost compared with the reference group, whereas an exp.(β) less than 1 represented a lower adjusted hospital cost. Incidence rate ratio (IRR) along with 95% CIs were used to report the results of the LOS. An IRR greater than 1 represented a longer adjusted LOS compared with the reference group, whereas an IRR less than 1 represented a shorter adjusted LOS.

All multivariable models adjusted for age, sex, race, residential income quartile, expected primary payer, hospital region, hospital bed size, hospital location/teaching status, residence type, and selected comorbidities. Reference categories for categorical variables are presented separately in Tables 3, 4, 5. Calendar year was modelled as a categorical variable, with 2017 serving as the reference year, to accommodate potentially nonlinear temporal patterns.

All tests were two‐sided, and a *p*‐value < 0.05 was considered statistically significant. Statistical analyses were performed using R version 4.5.2 (R Foundation for Statistical Computing, Vienna, Austria). R is an open‐source environment for statistical computing and graphics, and complex survey analyses were performed using survey‐weighted procedures appropriate for stratified, clustered, and weighted data.

## 3 Results

The analysis included 330,910 weighted adult hospitalizations with a primary diagnosis of hypoglycemia in the United States from 2017 to 2022. The cohort had a mean age of 67 years. Women represented 52% of admissions. White individuals formed the largest racial group at 53%, followed by Black individuals (28%) and Hispanic individuals (12%). Admissions were concentrated in the South and in urban teaching hospitals, which accounted for 44% and 70% of admissions, respectively. Medicare covered most admissions, with 230,915 (70%) hospitalizations. T2DM was present in 91% of admissions, CKD in 47%, hyperlipidemia in 53%, hypertension in 40%, AF in 18%, and obesity in 17%. The median LOS was 3.0 days (IQR, 2.0 to 5.0 days). Overall, 5300 admissions ended in in‐hospital death (1.6%) (Tables 1 and 2; Figures 1 and 2).

**TABLE 1:** Baseline characteristics and inpatient outcomes of adults hospitalized with primary hypoglycemia, 2017–2022.

Characteristic	*N* = 330,910^a^
Age, years	67 (15)
Race	
White	171,900 (53%)
Asian or Pacific Islander	10,495 (3.2%)
Black	91,230 (28%)
Hispanic	39,785 (12%)
Native American	2380 (0.7%)
Other	9110 (2.8%)
Sex	
Male	158,605 (48%)
Female	172,280 (52%)
Residential income	
Q1	118,640 (36%)
Q2	85,400 (26%)
Q3	70,700 (22%)
Q4	50,925 (16%)
Expected primary payer	
Medicaid	43,265 (13%)
Medicare	230,915 (70%)
Other	16,145 (4.9%)
Private	40,175 (12%)
Hospital region	
Midwest	66,915 (20%)
Northeast	64,310 (19%)
South	144,840 (44%)
West	54,845 (17%)
Hospital location and teaching status	
Urban, teaching	230,725 (70%)
Rural	30,740 (9.3%)
Urban, non‐teaching	69,445 (21%)
Hospital bedsize	
Large	147,220 (44%)
Medium	103,415 (31%)
Small	80,275 (24%)
Chronic kidney disease	154,180 (47%)
Congestive heart failure	17,845 (5.4%)
Iron deficiency anaemia	18,405 (5.6%)
Diabetes mellitus	300,025 (91%)
Smoking	37,145 (11%)
Hypertension	131,695 (40%)
Alcohol abuse	16,760 (5.1%)
HIV	2180 (0.7%)
Previous MI	27,030 (8.2%)
Atrial fibrillation	60,300 (18%)
Obesity	57,455 (17%)
Hyperlipidemia	175,570 (53%)

^a^ Mean (SD); *n* (%).

**TABLE 2:** Inpatient outcomes among adults hospitalized with primary hypoglycemia, 2017–2022.

Characteristic	*N* = 330,910^a^
In‐hospital mortality	5300 (1.6%)
Inflation‐adjusted total cost ($)	7319 (4728, 11,719)
Length of stay (days)	3.0 (2.0, 5.0)

^a^ *n* (%); median (Q1, Q3).

**FIGURE 1:**
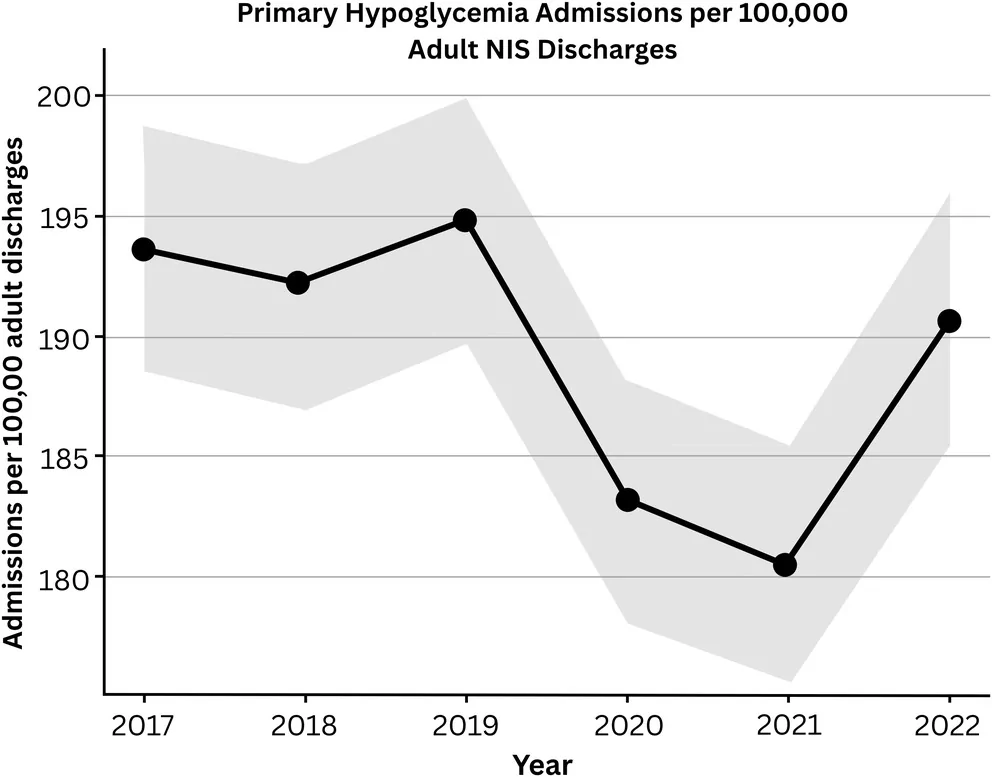
Primary hypoglycemia admissions per 100,000 adult NIS discharges, 2017–2022.

**FIGURE 2:**
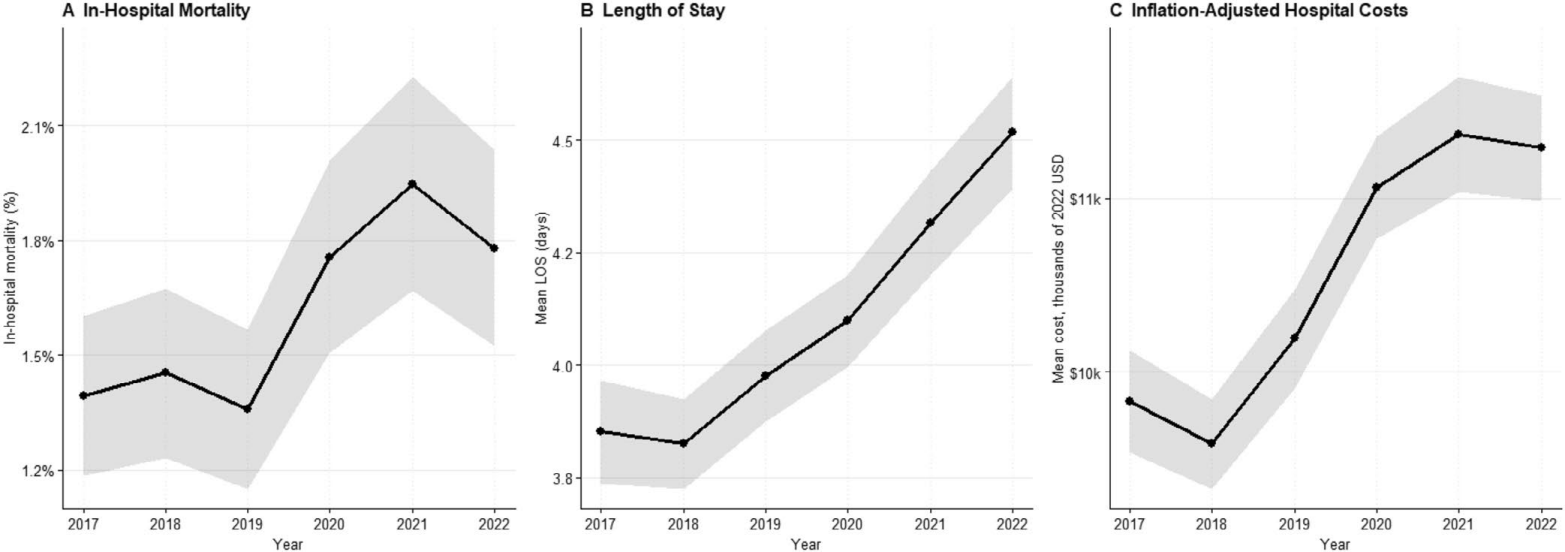
Trends in inpatient outcomes among primary hypoglycemia admissions, 2017–2022.

A non‐linear national admission trend was seen across the study period. Primary hypoglycemia admissions peaked near 2019 at about 195 discharges per 100,000 adult NIS discharges, then declined during 2020 and 2021, and partially increased again in 2022. Inpatient mortality rose after 2019 and reached its highest point around 2021. Mean LOS increased steadily from about 3.9 days in 2017 to about 4.5 days in 2022, though not in a strictly linear pattern. Higher inpatient cost levels were also observed over time, with mean inflation‐adjusted costs rising from under $10,000 in 2017 to above $11,000 by 2021–2022 (Tables 1 and 2; Figures 1 and 2).

### 3.1 Adjusted Mortality Model

In the adjusted mortality model, compared with 2017, the odds of in‐hospital death were significantly higher in 2020 (OR 1.30, 95% CI 1.04 to 1.61; *p* = 0.019), 2021 (OR 1.44, 95% CI 1.16 to 1.79; *p* = 0.001), and 2022 (OR 1.33, 95% CI 1.07 to 1.65; *p* = 0.011). Age also showed a significant positive association with mortality, with each additional year linked to 2% higher odds (OR 1.02, 95% CI 1.01 to 1.02; *p* < 0.001). Women showed lower adjusted odds of mortality than men (OR 0.80, 95% CI 0.70 to 0.91; *p* < 0.001). Small hospital bed size was associated with lower mortality odds compared with large hospitals (OR 0.76, 95% CI 0.65 to 0.90; *p* = 0.001). However, patient race and residential income quartiles were not independently associated with odds of in‐hospital mortality. Similarly, expected primary insurance provider type did not act as a significant independent predictor of death during hospitalization (Table 3).

**TABLE 3:** Multivariable predictors of in‐hospital mortality among adults hospitalized with primary hypoglycemia.

Characteristic	OR	95% CI	*p*
Year			
2017	—	—	
2018	1.07	0.86, 1.34	0.5
2019	0.96	0.76, 1.20	0.7
2020	1.30	1.04, 1.61	0.019
2021	1.44	1.16, 1.79	0.001
2022	1.33	1.07, 1.65	0.011
Age, years	1.02	1.01, 1.02	< 0.001
Sex			
Male	—	—	
Female	0.80	0.70, 0.91	< 0.001
Hospital location and teaching status			
Urban, teaching	—	—	
Rural	0.82	0.59, 1.13	0.2
Urban, non‐teaching	0.92	0.78, 1.08	0.3
Race			
White	—	—	
Asian or Pacific Islander	1.07	0.76, 1.51	0.7
Black	1.10	0.93, 1.29	0.3
Hispanic	0.79	0.61, 1.01	0.056
Native American	1.15	0.52, 2.55	0.7
Other	0.78	0.51, 1.21	0.3
Residential income			
Q1	—	—	
Q2	0.95	0.80, 1.12	0.5
Q3	0.97	0.81, 1.17	0.8
Q4	1.21	0.99, 1.49	0.064
Expected primary payer			
Medicaid	—	—	
Medicare	0.81	0.65, 1.03	0.082
Other	0.71	0.49, 1.02	0.067
Private	0.84	0.64, 1.09	0.2
Hospital region			
Midwest	—	—	
Northeast	1.07	0.88, 1.31	0.5
South	1.01	0.85, 1.20	> 0.9
West	1.03	0.83, 1.29	0.8
Hospital bedsize			
Large	—	—	
Medium	0.92	0.79, 1.06	0.2
Small	0.76	0.65, 0.90	0.001
Residence			
Large metro	—	—	
Micropolitan	1.50	1.13, 2.00	0.006
Small metro	1.23	1.06, 1.43	0.007
Chronic kidney disease			
No	—	—	
Yes	0.77	0.64, 0.92	0.005
Congestive heart failure			
No	—	—	
Yes	2.92	2.42, 3.54	< 0.001
Iron deficiency anaemia			
No	—	—	
Yes	1.14	0.89, 1.47	0.3
Diabetes mellitus			
No	—	—	
Yes	0.57	0.47, 0.69	< 0.001
Smoking			
No	—	—	
Yes	1.20	0.98, 1.47	0.078
Hypertension			
No	—	—	
Yes	0.46	0.38, 0.56	< 0.001
Alcohol abuse			
No	—	—	
Yes	0.91	0.67, 1.25	0.6
HIV			
No	—	—	
Yes	1.48	0.75, 2.92	0.3
Previous MI			
No	—	—	
Yes	0.91	0.72, 1.15	0.5
Atrial fibrillation			
No	—	—	
Yes	1.51	1.29, 1.76	< 0.001
Obesity			
No	—	—	
Yes	0.74	0.61, 0.90	0.002
Hyperlipidemia			
No	—	—	
Yes	0.63	0.55, 0.72	< 0.001

Abbreviations: CI, confidence interval; OR, odds ratio.

Patients from micropolitan areas had higher mortality odds than those from large metropolitan areas (OR 1.50, 95% CI 1.13 to 2.00; *p* = 0.006). Similarly, patients from small metropolitan areas also had higher mortality odds (OR 1.23, 95% CI 1.06 to 1.43; *p* = 0.007) comparatively. CHF showed highest mortality, with nearly three‐fold higher odds of death (OR 2.92, 95% CI 2.42 to 3.54; *p* < 0.001). Lower mortality odds were observed among patients with CKD (OR 0.77, 95% CI 0.64 to 0.92; *p* = 0.005) (Table 3).

AF was associated with increased mortality (OR 1.51, 95% CI 1.29 to 1.76; *p* < 0.001). Diabetes (OR 0.57, 95% CI 0.47 to 0.69; *p* < 0.001), hypertension (OR 0.46, 95% CI 0.38 to 0.56; *p* < 0.001), obesity (OR 0.74, 95% CI 0.61 to 0.90; *p* = 0.002) and hyperlipidemia (0.63, 95% CI 0.55 to 0.72; *p* < 0.001) were associated with lower mortality odds (Table 3).

### 3.2 Length of Stay in Hospital

LOS was higher in 2020, 2021, and 2022 compared with 2017 (2020: IRR 1.05, 95% CI 1.01 to 1.09; *p* = 0.020; 2021: IRR 1.11, 95% CI 1.06 to 1.15; *p* < 0.001; and 2022: IRR 1.16, 95% CI 1.12 to 1.21; *p* < 0.001). Conversely, advanced patient age was not significantly associated with changes in the duration of hospital stays (IRR 1.00, 95% CI 1.00 to 1.00; *p* = 0.9). Patient sex was similarly non‐significant (IRR 0.99, 95% CI 0.97 to 1.01; *p* = 0.4). Urban teaching hospitals had longer stays than rural hospitals (IRR 1.25, 95% CI 1.21 to 1.30; *p* < 0.001). Similarly, urban non‐teaching hospitals also had longer stays (IRR 1.10, 95% CI 1.05 to 1.14; *p* < 0.001). Shorter LOS was also seen among Hispanic individuals compared with White individuals (IRR 0.94, 95% CI 0.90 to 0.97; *p* < 0.001) (Table 4).

**TABLE 4:** Multivariable predictors of length of stay among adults hospitalized with primary hypoglycemia.

Characteristic	IRR	95% CI	*p*
Year			
2017	—	—	
2018	0.99	0.95, 1.03	0.7
2019	1.02	0.98, 1.06	0.3
2020	1.05	1.01, 1.09	0.020
2021	1.11	1.06, 1.15	< 0.001
2022	1.16	1.12, 1.21	< 0.001
Age, years	1.00	1.00, 1.00	0.9
Sex			
Female	—	—	
Male	0.99	0.97, 1.01	0.4
Race			
White	—	—	
Asian or Pacific Islander	1.00	0.94, 1.05	0.9
Black	1.11	1.08, 1.13	< 0.001
Hispanic	0.94	0.90, 0.97	< 0.001
Native American	0.98	0.88, 1.09	0.7
Other	0.96	0.89, 1.03	0.2
Residential income			
Q1	—	—	
Q2	1.01	0.98, 1.03	0.7
Q3	0.96	0.93, 0.99	0.004
Q4	0.96	0.93, 0.99	0.020
Expected primary payer			
Medicaid	—	—	
Medicare	0.96	0.92, 1.00	0.056
Other	0.89	0.82, 0.96	0.004
Private	0.90	0.86, 0.95	< 0.001
Hospital region			
Midwest	—	—	
Northeast	1.11	1.07, 1.15	< 0.001
South	1.08	1.05, 1.10	< 0.001
West	0.94	0.91, 0.98	0.001
Hospital Location and Teaching status			
Rural	—	—	
Urban, non‐teaching	1.10	1.05, 1.14	< 0.001
Urban, teaching	1.25	1.21, 1.30	< 0.001
Hospital bedsize			
Large	—	—	
Medium	0.89	0.87, 0.92	< 0.001
Small	0.83	0.81, 0.85	< 0.001
Chronic kidney disease			
No	—	—	
Yes	1.03	0.99, 1.07	0.13
Congestive heart failure			
No	—	—	
Yes	1.64	1.57, 1.71	< 0.001
Iron deficiency anaemia			
No	—	—	
Yes	1.25	1.20, 1.31	< 0.001
Diabetes mellitus			
No	—	—	
Yes	0.93	0.89, 0.96	< 0.001
Smoking			
No	—	—	
Yes	0.94	0.90, 0.98	0.003
Hypertension			
No	—	—	
Yes	0.89	0.85, 0.92	< 0.001
Alcohol abuse			
No	—	—	
Yes	1.07	1.00, 1.15	0.046
HIV			
No	—	—	
Yes	1.04	0.85, 1.28	0.7
Previous MI			
No	—	—	
Yes	0.95	0.92, 0.98	0.005
Atrial fibrillation			
No	—	—	
Yes	1.11	1.08, 1.13	< 0.001
Obesity			
No	—	—	
Yes	1.02	1.00, 1.05	0.091
Hyperlipidemia			
No	—	—	
Yes	0.91	0.90, 0.93	< 0.001

Abbreviations: CI, confidence interval; IRR, incidence rate ratio.

Black individuals were associated with longer adjusted hospital stay compared with White individuals (IRR 1.11, 95% CI 1.08 to 1.13; *p* < 0.001). Patients in the third income quartile had slightly shorter stays than those in the lowest quartile (IRR 0.96, 95% CI 0.93 to 0.99; *p* = 0.004). Compared with Medicaid, other payer status (IRR 0.89, 95% CI 0.82 to 0.96; *p* = 0.004) and private insurance (IRR 0.90, 95% CI 0.86 to 0.95; *p* < 0.001) were associated with shorter stays. The association for Medicare was not statistically significant (IRR 0.96, 95% CI 0.92 to 1.00; *p* = 0.056) (Table 4).

Regional differences were also identified in the adjusted model. Compared with the Midwest, the Northeast (IRR 1.11, 95% CI 1.07 to 1.15; *p* < 0.001) and the South had longer stays (IRR 1.08, 95% CI 1.05 to 1.10; *p* < 0.001). The stays were short in the West comparatively (IRR 0.94, 95% CI 0.91 to 0.98; *p* = 0.001). Medium‐sized hospitals (IRR 0.89, 95% CI 0.87 to 0.92; *p* < 0.001) and small hospitals showed shorter stays (IRR 0.83, 95% CI 0.81 to 0.85; *p* < 0.001) as compared to large size hospitals (Table 4).

Comorbid illness was associated with LOS. CHF was positively associated with LOS (IRR 1.64, 95% CI 1.57 to 1.71; *p* < 0.001) followed by iron deficiency anaemia (IRR 1.25, 95% CI 1.20 to 1.31; *p* < 0.001), and AF (IRR 1.11, 95% CI 1.08 to 1.13; *p* < 0.001). Several comorbidities were associated with shorter adjusted stays. Diabetes mellitus was associated with shorter LOS (IRR 0.93, 95% CI 0.89 to 0.96; *p* < 0.001). Smoking was also associated with shorter stay (IRR 0.94, 95% CI 0.90 to 0.98; *p* = 0.003). Hypertension, previous myocardial infarction, and hyperlipidemia were associated with shorter stays, with IRR values of 0.89 (95% CI 0.85 to 0.92; *p* < 0.001), 0.95 (95% CI 0.92 to 0.98; *p* = 0.005), and 0.91 (95% CI 0.90 to 0.93; *p* < 0.001), respectively (Table 4).

### 3.3 Inflation‐Adjusted Hospital Cost

Inflation‐adjusted hospital costs were higher in 2020, 2021, and 2022 compared with 2017 (2020: exp.(β) 1.12, 95% CI 1.08 to 1.16; *p* < 0.001; 2021: exp.(β) 1.15, 95% CI 1.11 to 1.20; *p* < 0.001; and 2022: exp.(β) 1.15, 95% CI 1.11 to 1.19; *p* < 0.001). Age was significantly associated with cost, although the rounded effect estimate was 1.00 (95% CI 1.00 to 1.00; *p* < 0.001). Men had higher costs than women (exp(β) 1.02, 95% CI 1.00 to 1.04; *p* = 0.017). Compared with rural hospitals, lower costs were observed in urban non‐teaching hospitals (exp(β) 0.96, 95% CI 0.92 to 0.99; *p* = 0.010), whereas higher costs were observed in urban teaching hospitals (exp(β) 1.06, 95% CI 1.02 to 1.09; *p* < 0.001). Race showed several significant cost differences. Asian or Pacific Islander individuals had higher adjusted costs than White individuals (exp(β) 1.10, 95% CI 1.04 to 1.15; *p* < 0.001). Black individuals also had higher adjusted costs (exp(β) 1.12, 95% CI 1.09 to 1.15; *p* < 0.001). Patients in the highest residential income quartile had higher costs than those in the lowest quartile (exp(β) 1.11, 95% CI 1.07 to 1.14; *p* < 0.001). A significant cost reduction was seen for patients with other payer status compared with Medicaid (exp(β) 0.90, 95% CI 0.84 to 0.96; *p* < 0.001) (Table 5).

**TABLE 5:** Multivariable predictors of inflation‐adjusted hospital cost among adults hospitalized with primary hypoglycemia.

Characteristic	Exp (Beta)	95% CI	*p*
Year			
2017	—	—	
2018	0.98	0.95, 1.02	0.3
2019	1.03	0.99, 1.06	0.2
2020	1.12	1.08, 1.16	< 0.001
2021	1.15	1.11, 1.20	< 0.001
2022	1.15	1.11, 1.19	< 0.001
Age, years	1.00	1.00, 1.00	< 0.001
Sex			
Female	—	—	
Male	1.02	1.00, 1.04	0.017
Race			
White	—	—	
Asian or Pacific Islander	1.10	1.04, 1.15	< 0.001
Black	1.12	1.09, 1.15	< 0.001
Hispanic	1.03	0.99, 1.06	0.14
Native American	0.99	0.89, 1.10	0.9
Other	1.09	1.02, 1.16	0.013
Residential income			
Q1	—	—	
Q2	1.03	1.00, 1.06	0.020
Q3	1.03	1.00, 1.05	0.063
Q4	1.11	1.07, 1.14	< 0.001
Expected primary payer			
Medicaid	—	—	
Medicare	0.98	0.94, 1.01	0.2
Other	0.90	0.84, 0.96	< 0.001
Private	0.96	0.92, 1.01	0.094
Hospital region			
Midwest	—	—	
Northeast	1.18	1.14, 1.22	< 0.001
South	0.93	0.91, 0.96	< 0.001
West	1.27	1.23, 1.32	< 0.001
Hospital location and teaching status			
Rural	—	—	
Urban, non‐teaching	0.96	0.92, 0.99	0.010
Urban, teaching	1.06	1.02, 1.09	< 0.001
Hospital bedsize			
Large	—	—	
Medium	0.92	0.90, 0.95	< 0.001
Small	0.90	0.88, 0.93	< 0.001
Chronic kidney disease			
No	—	—	
Yes	1.07	1.03, 1.10	< 0.001
Congestive heart failure			
No	—	—	
Yes	1.64	1.58, 1.71	< 0.001
Iron deficiency anaemia			
No	—	—	
Yes	1.24	1.19, 1.28	< 0.001
Diabetes mellitus			
No	—	—	
Yes	0.93	0.90, 0.96	< 0.001
Smoking			
No	—	—	
Yes	0.98	0.95, 1.01	0.2
Hypertension			
No	—	—	
Yes	0.91	0.88, 0.94	< 0.001
Alcohol abuse			
No	—	—	
Yes	1.07	1.01, 1.12	0.014
HIV			
No	—	—	
Yes	1.06	0.93, 1.20	0.4
Previous MI			
No	—	—	
Yes	0.97	0.94, 1.00	0.040
Atrial fibrillation			
No	—	—	
Yes	1.13	1.10, 1.15	< 0.001
Obesity			
No	—	—	
Yes	1.04	1.02, 1.06	0.001
Hyperlipidemia			
No	—	—	
Yes	0.93	0.91, 0.95	< 0.001

Abbreviation: CI, confidence interval.

Compared with the Midwest, the West had the largest regional increase in cost (exp(β) 1.27, 95% CI 1.23 to 1.32; *p* < 0.001) followed by the Northeast (exp(β) 1.18, 95% CI 1.14 to 1.22; *p* < 0.001). The South had lower adjusted costs (exp(β) 0.93, 95% CI 0.91 to 0.96; *p* < 0.001). Moreover, hospital bed size also showed a strong association with cost. Medium‐sized hospitals had lower costs than large hospitals (exp(β) 0.92, 95% CI 0.90 to 0.95; *p* < 0.001). Small hospitals also had lower costs (exp(β) 0.90, 95% CI 0.88 to 0.93; *p* < 0.001) (Table 5).

CHF was associated with the largest cost increase in the full model (exp(β) 1.64, 95% CI 1.58 to 1.71; *p* < 0.001) followed by iron deficiency anaemia (exp(β) 1.24, 95% CI 1.19 to 1.28; *p* < 0.001), AF (exp(β) 1.13, 95% CI 1.10 to 1.15; *p* < 0.001), alcohol abuse (exp(β) 1.07, 95% CI 1.01 to 1.12; *p* = 0.014), CKD (exp(β) 1.07, 95% CI 1.03 to 1.10; *p* < 0.001) and obesity (exp(β) 1.04, 95% CI 1.02 to 1.06; *p* = 0.001). Several diagnoses showed lower adjusted hospital costs. Diabetes mellitus was associated with lower cost (exp(β) 0.93, 95% CI 0.90 to 0.96; *p* < 0.001). Hypertension was associated with lower costs (exp(β) 0.91, 95% CI 0.88 to 0.94; *p* < 0.001). Previous myocardial infarction and hyperlipidemia were also linked to lower costs, with exp.(β) values of 0.97 (95% CI 0.94 to 1.00; *p* = 0.040) and 0.93 (95% CI 0.91 to 0.95; *p* < 0.001), respectively (Table 5).

## 4 Discussion

The analysis included 330,910 weighted adult hospitalizations with a primary diagnosis of hypoglycemia in the United States from 2017 to 2022. Admissions were mostly among older adults, with a mean age of 67 years. More than 90% of our hospitalized population was diabetic, followed by most patients who were insured by Medicare, and the burden was concentrated in patients with diabetes, CKD, hypertension, hyperlipidemia, AF, and obesity. Primary hypoglycemia admissions peaked around 2019, declined during 2020–2021, and partially increased again in 2022; however, inpatient mortality, LOS, and inflation‐adjusted hospital costs increased over time, though non‐linearly, with mortality peaking around 2021. In adjusted analyses, compared with 2017, mortality odds, LOS, and hospital costs were significantly higher during several later years, particularly from 2020 through 2022, although the yearly patterns were nonlinear. CHF and AF showed the most consistent adverse associations, as both increased mortality, LOS, and cost, while iron deficiency anaemia, CKD, alcohol abuse, and obesity mainly increased resource use. Overall, advanced age, micropolitan or small metropolitan residence, CHF, and AF emerged as the primary independent predictors of worse clinical outcomes, with cardiovascular comorbidities carrying the highest mortality burden and prolonged healthcare utilization.

Our study found a significant association of the age, gender, residential status, and hospital bedsize with the mortality. Increasing age was associated with higher odds of death. Similarly, men and patients from micropolitan or small metropolitan areas had higher mortality odds, whereas patients admitted to small hospitals had lower mortality odds, likely reflecting case selection or transfer of more complex cases to larger hospitals. According to our study, age was a strong predictor (*p* < 0.001), as literature suggests, hypoglycemia is usually associated with chronic conditions and older age, which is often accompanied by high comorbidity burden, and frailty [18]. According to McCoy RG et al., multicomorbidity had a significant association with hypoglycemia hospitalizations, as they found, the IRR increased by 148% from 1.66 for at least 2 comorbidities to 4.12 for eight or more comorbid conditions [7]. Similarly, patients aged > 75 years had 58% higher IRRs of hospitalizations compared to < 45‐year age group. It is vastly supported by the medical literature [19, 20, 21]. Gender related differences in hypoglycemia is not well explored in the literature. Studies show contradictory results with inconclusive evidence. A study done back in 2020 showed women had lower risk of hypoglycemia related hospitalization (HR 0.73; 95% CI 0.54 to 0.98) [22]; similarly, another study noted that women with T1DM showed lower decline in glucose level after physical activities compared to men, thus, showing a comparatively balanced glucose profile [23]. Another study by Brockman et al. showed men were more prone to hypoglycemia [24]. In contrast, larger cohort studies did not find any meaningful association [7, 25]. Moreover, high mortality among micropolitan and small metropolitan can be explained by delayed access to care, fewer endocrinologists, fewer diabetes education services, weaker emergency access, longer travel times, and less continuity after discharge [26, 27, 28]. Similarly, lower odds of deaths at hospitals with small bed size can be explained based on the case selection of the cases. As these facilities are not well equipped to manage more complex cases thus, such cases are transferred to larger hospitals [29]. In addition, our study reported that CHF or AF patients with hypoglycemia related hospitalizations showed higher odds of mortality. Although diabetes, CKD, hyperlipidemia, and hypertension were more prevalent in our cohort, CHF and AF were associated with the highest mortality, likely because these conditions identify a smaller but clinically more fragile subgroup with limited cardiovascular reserve. In such patients, hypoglycemia may act as an acute physiologic stressor, provoking sympathetic activation, arrhythmogenic instability, hemodynamic compromise, and decompensation of underlying cardiac disease. Therefore, the higher mortality observed in CHF and AF patients may reflect severity of baseline cardiovascular vulnerability rather than the overall prevalence of these comorbidities in the cohort. Literature is well settled on the fact that CHF individuals are frail and can develop cardiometabolic adversities, and have higher chances of suffering from hypoglycemia during hospitalizations [30]. A study by Cha et al. found that T2DM and hypoglycemia had 2.6 times higher chances of all‐cause mortality, especially when hospitalized due to HF [30]. Similar conclusion is supported by another study [31]. In the same manner, multiple studies support the relation of AF and hypoglycemia, that is, sever hypoglycemic population is known to be prone to AF [32, 33]. Older age, men, and micropolitan or small metropolitan residence were linked with higher mortality after hypoglycemia hospitalization, likely because these patients have more frailty, comorbidities, delayed access to care, and weaker follow‐up services. Women may have shown lower mortality due to differences in case mix or glucose regulation, but the literature remains mixed and does not prove a clear protective effect. CHF and AF further increased mortality risk, suggesting that hypoglycemia becomes more dangerous when it occurs in patients with poor cardiovascular reserve. Diabetes, hypertension, hyperlipidemia, obesity, and CKD were associated with lower adjusted mortality odds. These findings should be interpreted with caution rather than as evidence of a protective mechanism. Patients with these chronic conditions who were hospitalized primarily for hypoglycemia may represent a comparatively lower‐acuity subgroup than those whose hypoglycemia was secondary to more severe illness, and differential comorbidity coding in administrative data may further contribute. Similar paradoxical inverse associations between common cardiometabolic comorbidities and short‐term mortality have been reported elsewhere and are generally attributed to confounding by indication or selection rather than true biological protection.

Several programs were launched in the last decade in order to prevent severe hypoglycemia related adverse events. One such program was national action plan for adverse event prevention by US Department of Health and Human Services [34]. It was launched back in 2014 to prevent adverse events of the diabetes agents. Another similar program was Avalere Hypoglycemia Prevention Initiative, launched in 2017 and helped reduce over intake of hypoglycemic medications [35]. Similarly, other programs were launched in order to spread awareness among the people in order to control such events [36, 37]. However, limited national‐level data are available to determine how successful these programs were as their implementation was not entirely national, but was rather segmented. Even though US improved in its diabetes related care but its system is fragmented, that is, even the Diabetes Self‐Management Education and Support system is merely used by 5% of the Medicare beneficiaries during the first year of diabetes diagnosis [36, 38]. However, in comparison, Denmark and UK have well‐established nationwide programs. The Danish Adult Diabetes Registry [39], operates on the national level, linking data, thus making tracking of hypoglycemia easier across the whole country. Their rate of hospitalization for hypoglycemia fell by more than 60% for diabetes between 1998 and 2018 [40]. Similarly, National Diabetes Inpatient Audit (NaDIA) [41] keeps track of the hypoglycemia episodes in the inpatients. Following implementation of NaDIA and related inpatient diabetes safety guidance, severe hypoglycemic episodes declined from 12% of inpatients with diabetes in 2010 to 7% in 2019, while use of injectable rescue treatment fell from 2.4% to 1.4% [42]. On the other hand, COVID‐19 disrupted the rehabilitation centers that were managing the burden of chronic diseases like HF, AF, and CKD. According to the reports, during the early outbreak of the pandemic, participation of the HF patients at outpatient clinics, cardiac rehabilitation centers, and remote monitoring systems fell drastically [43]. Similarly, another study reported the rapid transition to a telehealth‐based model altered standard care processes, with 88.5% of the HF outpatient visits in early 2020 conducted via telehealth [44]. This disruption lead to reduced care delivery to the required patients. Therefore, the decline in hypoglycemia admissions during 2020–2021 in our study may reflect reduced care‐seeking and disrupted outpatient management rather than a true fall in disease severity, while the later rise and worsening mortality, LOS, and cost may reflect the accumulated effect of delayed CHF, AF, CKD, and diabetes care in a population whose chronic cardiometabolic burden was already increasing. Thus, the United States had several hypoglycemia‐prevention programs, but their impact was limited by fragmented implementation and low use of diabetes self‐management services. Denmark and the UK had stronger national tracking systems, which helped reduce hypoglycemia hospitalizations and inpatient severe hypoglycemic events over time. COVID‐19 disrupted chronic disease care for CHF, AF, CKD, and diabetes, so the 2020–2021 decline may reflect reduced care‐seeking, while the later rise may reflect delayed and accumulated disease burden.

Based on the inflation adjusted cost analysis of our study, CHF showed the highest cost increase followed by iron deficiency anaemia and AF. Black and Asian race, hospitals with teaching status, and with a large bed size showed higher costs comparatively. Similarly, West and Northeast showed the highest increase in the cost. Our findings lie in parallel with the current literature, as Osenenko et al. report that cost per HF hospitalization ranged from a mean of $10,737 to $17,830; similarly, charges per hospital admission of HF ranged from a mean of $50,569 to $50,952 [45]. Similarly, AF was associated with incremental annual medical costs of $11,393. Outpatient care contributed to a total of $7059 for services while $1875 were contributed by prescriptions [46]. A JAMA study found that Black individuals had 19% higher inpatient spending and 12% higher emergency department spending than the all‐population mean, despite 26% lower ambulatory‐care spending [46, 47]. This lines in parallel to our findings; however, Asian, Native Hawaiian, and Pacific Islander individuals had lower total age‐standardized spending which was in contrast to our results. A likely explanation can be disease‐specific and inpatient‐specific results, reflecting case mix, hospital region, hospital type, or admission severity among the patients hospitalized specifically for hypoglycemia, rather than general national spending patterns. Moreover, a report by Jiang et al. [48] demonstrates that rural hospital stays were shorter and less expensive than urban stays, with average costs of $6500 in rural hospitals vs. $9000 in urban hospitals. In addition, average costs at large urban hospitals were 48.9% higher than at small urban hospitals, or $27,100 vs. $18,200. They further found that even after wage‐index adjustment, Pacific charges stayed above the national mean at 1.14 and Middle Atlantic at 1.08; thus, demonstrating that West had relatively higher costs. Finally, CHF, iron deficiency anaemia, and AF were the main drivers of higher inflation‐adjusted hospital costs in our study, consistent with prior evidence showing high inpatient costs for HF and substantial incremental spending with AF. Higher costs among Black patients aligned with national data showing greater inpatient and ED spending, while the higher cost among Asian patients may reflect hypoglycemia‐specific case mix rather than general spending trends. Urban teaching hospitals, large hospitals, and hospitals in the West and Northeast also had higher costs, likely due to greater case complexity, higher resource use, and regional price differences.

Future studies should investigate how a unified national hypoglycemia surveillance and prevention system can be integrated into the currently fragmented US diabetes‐care structure. A registry‐linked model, similar to systems used in Denmark or the UK, may help track high‐risk patients, standardize reporting, and guide prevention across outpatient and inpatient settings. Our findings have important policy implications for identifying patients at high risk of hypoglycemia‐related hospitalization, especially older adults and those with CHF, AF, CKD, anaemia, alcohol use, and obesity. Future policies should focus on earlier risk detection, stronger outpatient monitoring, and better coordination between diabetes, cardiovascular, and renal care systems.

However, some limitations must be kept in mind before interpreting the data. One, that the current claims rely on the data extracted from the administrative discharge data which only stores the severe events that were referred to the hospital; however, hypoglycemic events that did not result in hospitalizations or no visits to the hospitals could not be accounted for [49, 50, 51]. Second, clinical parameters like HbA1c, blood glucose levels, and other baselines could not be accounted for [52]. Third, medication‐related records could not be analysed that could directly impact the reasons for hospitalizations. Fourth, the retrospective design prevents causal interpretation, and the observed associations should be viewed as markers of risk rather than proof that these factors directly caused mortality, longer stay, or higher cost. Fifth, although calendar year was modelled categorically to accommodate nonlinear temporal patterns, the six‐year study period limits more detailed characterization of temporal changes. Sixth, hospital cost and LOS were right‐skewed; therefore, cost was modelled using a survey‐weighted GLM with a gamma distribution and log link, while LOS was modelled using a survey‐weighted GL with a quasi‐Poisson distribution and log link.

## 5 Conclusion

Primary hypoglycemia hospitalizations in the United States showed a fluctuating pattern from 2017 to 2022, with a decline during 2020–2021 followed by a partial rebound in 2022. Despite this decline, mortality, LOS, and inflation‐adjusted costs increased over time, suggesting greater clinical complexity among hospitalized patients. Older age, men, micropolitan or small metropolitan residence, CHF, and AF identified higher‐risk groups. CHF, iron deficiency anaemia, and AF were major drivers of resource use. These findings support stronger outpatient monitoring, integrated cardiometabolic care, and national surveillance to reduce preventable severe hypoglycemia events nationally in the future.

## Author Contributions

**Abu Huraira Bin Gulzar:** conceptualization, methodology, investigation, writing – original draft, writing – review and editing, project administration, resources, supervision. **Sheraz Hakeem:** conceptualization, methodology, investigation, writing – original draft. **Muhammad Umer Sohail:** methodology, formal analysis, supervision, project administration, resources, software. **Aima Tahir:** writing – original draft, visualization, software, data curation. **Tamseer Tariq:** writing – original draft, writing – review and editing, investigation, methodology, data curation. **Rasheeda Shehroz:** methodology, writing – original draft, writing – review and editing, visualization. **Moiz Shah Khan:** methodology, writing – original draft, writing – review and editing, software. **Ismail Rahim:** writing – original draft, writing – review and editing, investigation. **Umme Aiman:** validation, writing – review and editing, writing – original draft, visualization. **Muhammad Afnan Ashraf:** visualization, writing – original draft, investigation. **Shanzey Rai:** writing – original draft, writing – review and editing, investigation, validation. **Marriam Mavora:** writing – original draft, writing – review and editing, investigation, validation. **Asad Ali Ahmed Cheema:** writing – original draft, writing – review and editing, investigation, validation. **Hina Nadeem:** writing – review and editing, writing – original draft.

## Funding

The authors have nothing to report.

## Ethics Statement

This study used de‐identified, publicly available administrative data and did not involve direct patient contact. Therefore, institutional review board approval was not required.

## Consent

Patient consent was not required because this study analysed de‐identified administrative data.

## Conflicts of Interest

The authors declare no conflicts of interest.

## Data Availability

The data used in this study are publicly available through the Healthcare Cost and Utilization Project National Inpatient Sample database. Restrictions apply to the availability of these data, which were used under licence for the present study.
